# Detailed description and illustration of larva, pupa and imago of *Holorusia mikado* (Westwood, 1876) (Diptera: Tipulidae) from Japan

**DOI:** 10.3897/BDJ.9.e58009

**Published:** 2021-05-01

**Authors:** Levente-Péter Kolcsár, Takeyuki Nakamura, Daichi Kato, Kozo Watanabe

**Affiliations:** 1 Center for Marine Environmental Studies (CMES), Ehime University, Matsuyama, Japan Center for Marine Environmental Studies (CMES), Ehime University Matsuyama Japan; 2 The Shirakami Research Center for Environmental Sciences, Faculty of Agriculture and Life Science, Hirosaki University, Hirosaki, Aomori, Japan The Shirakami Research Center for Environmental Sciences, Faculty of Agriculture and Life Science, Hirosaki University Hirosaki, Aomori Japan; 3 Echigo-Matsunoyama Museum of Natural Sciences, ‘Kyororo’, Tôkamachi, Japan Echigo-Matsunoyama Museum of Natural Sciences, ‘Kyororo’ Tôkamachi Japan

**Keywords:** anal field, biology, chaetotaxy, ecology, head capsule, ovipositor, pupal sheath, spiracular field

## Abstract

**Background:**

*Holorusia* Loew, 1863 (Diptera: Tipulidae) is a relatively large crane fly genus with a wide distribution in the Afrotropic, Australasian–Oceanian, Eastern Palearctic, Oriental and Nearctic Regions. Although the genus is well known to include the largest crane fly species, the immature stages are, thus far, only described for the larva and pupa of the North American *Holorusia hesperea* Arnaud & Byers, 1990.

**New information:**

In this study, we describe for the first time the egg, larva and pupae of the Japanese *Holorusia mikado* (Westwood, 1876). Larvae were collected from semi-aquatic habitats, from slow flowing areas of streams and small waterfalls where leaf litter accumulates; the larvae are detritivores and feed on wet, decomposing leaves. The larvae were reared to adults in the laboratory. Morphological characters of immature stages discussed with comparison with the North American *H. hespera*. Male and female genitalia are illustrated and described in detail for the first time.

## Introduction

*Holorusia* Loew, 1863 is a large genus of Tipulidae, with 115 species described thus far. The majority of *Holorusia* species are endemic to the Oriental Region (75 species), while the remaining species occur in the Australasian–Oceanian (20 species), Afrotropical (11 species), Eastern Palearctic (15 species) and Nearctic (one species) Regions (Oosterbroek 2020). Two *Holorusia* species have been reported so far in Japan: *Holorusia mikado* (Westwood, 1876) from the Honshu, Kyushu and Shikoku Islands and *Holorusia esakii* (Takahashi, 1960) from Amami Island (Takahashi 1960, Nakamura 2014, Oosterbroek 2020).

The genus includes the largest known crane fly species, with one specimen of "*Holorusia mikado*", holding The Guinness World Record (2018) as “the world largest specimen of crane fly belonging to the species *Holorusia mikado*” (https://www.guinnessworldrecords.com/).

Exact systematic position of the genus *Holorusia* is not clarified so far. Early studies suggested a close relationship between *Prionocera* Loew and *Holorusia*; however, it was questioned by Vane-Wright (1967). Phylogenetic analysis of combined molecular markers (28S rDNA and CAD) and morphological characters (adults, larvae and pupae), revealed a close relationship between the North-American *Holorusia hespera* andTipula (Nippotipula) abdominalis (Say) (Petersen et al. 2010).

The genus was revised by Vane-Wright (1967) and the following characteristics of the genus established: antenna 12-14 segmented, subserrate or filiform, with short verticils; nasus present or absent; R_4_ and R_5_ curved towards each other; axillary area of wing well developed, calypter bare; femora with terminal ctenidium (comb of black spike-like setae); male genitalia relatively simple; tergite 9 fleshy, usually deeply emarginate, with long and dense hairs; lobe of gonostylus (outer gonostylus) large, fleshy with fine, short hairs; clasper of gonostylus (inner gonostylus) also simple, dorsally curved, translucent rod, usually with divided tip; cercus strongly sclerotised, narrowed outwardly, with rounded tip; hypogynal valve moderate in length; genital fork Y-shaped with three spermathecae.

The immature stages of the genus are currently only described for Western North American *Holorusia hespera* Arnaud et Byers, 1990 (=*rubiginosa* Loew, 1863) (Alexander 1920, Gelhaus 2008, Gelhaus 1986).

We collected the undescribed larvae of *Holorusia mikado* from the Honshu and Shikoku Islands and reared them in our laboratory. Here, we describe and illustrate the last instar larva, the pupae of both sexes, habitus and terminalia of the imagos and the unfertilised egg. The morphology of the larva and pupa was compared with those of the Nearctic *H. hespera*.

## Materials and methods

**Figure 1.**
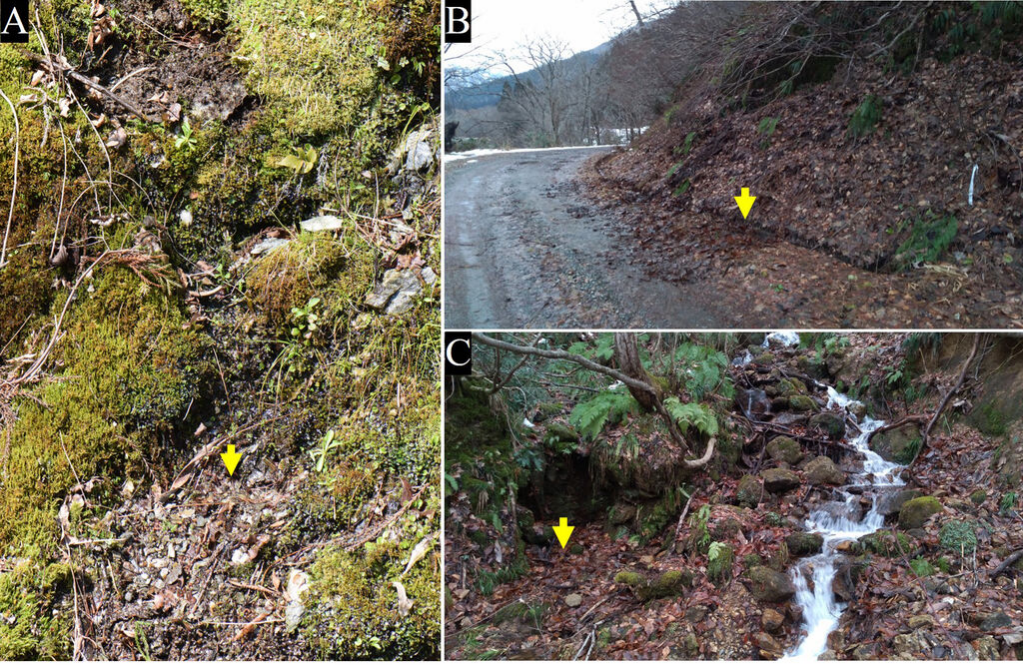
Habitats of *Holorusia mikado* (Westwood, 1876). **A.** Small spring with loose, gravelly soil rich in detritus in Japan, Kochi Pref., Tosa, Higashiishihara, alt. 705 m, 33°41.74'N 133°26.51'E; **B.** Drainage ditch at the side of a mountain road with accumulated litter; **C.** Bank of stream with accumulated litter in Japan, Yamagata Pref., Oguni, Arakawa River Valley, alt. 340 m, 38°11.56'N; 139°48.2'E. Yellow arrows indicate exact locations where larvae were collected.

Larvae were collected by hand from a drainage ditch at the side of the road, stream banks, around small waterfalls and small springs in Japan (Fig. 1). Larvae were reared in a 20 cm diameter, round box filled with substrate collected from the larval habitats. When larvae had consumed the decaying leaves, additional leaves were added and the substrate was kept wet.

Male and female terminalia and larva head capsules were photographed in glycerol, after maceration with 10-15% potassium hydroxide (KOH) at room temperature. Pupal exuviae were cleaned in soapy water to remove dirt before photographing. A few larvae were killed by boiling water and preserved in 90% ethanol. Photos were taken using a Zeiss Stemi 508 stereomicroscope, equipped with a Canon Kiss M digital camera. Stack photos were combined using Zerene Stacker software. Illustrations were made in Adobe Photoshop CC 2019. Drawings were created by Takeyuki Nakamura.

The terminology used for larval and pupal morphological features follows Brindle (1960) and the head capsule terminology follows Neugart et al. (2009). General terminology of the adult follows Cumming and Wood (2017). In the case of the wing venation, we followed the traditional venation system. The gonostylus terminology follows Ribeiro (2006).

**Depositories**

**CKLP** Private collection of L.-P. Kolcsár

**EMNS** Echigo-Matsunoyama Museum of Natural Sciences, Tôkamachi, Japan

**SCHU** The Shirakami Research Center, Hirosaki University, Japan

## Taxon treatments

### Holorusia mikado

(Westwood, 1876)

41DCFE7E-9EE6-57B6-9E1D-0C85A6F62F6A

Tipula mikado Westwood in Westwood 1876
Holurusia mikado (Westwood) in Enderlein 1917
Ctenacroscelis mikado (Westwood) in Edwards 1921
Holurusia mikado (Westwood) in Vane-Wright 1967

#### Materials

- **Type status:** Other material. **Occurrence:** recordedBy: L.-P. Kolcsár; individualCount: 4; sex: 2 males, 2 females; lifeStage: adult; **Location:** island: Honshu; country: Japan; stateProvince: Yamagata; municipality: Oguni; locality: Arakawa River Valley; verbatimElevation: 340 m; decimalLatitude: 38.192667; decimalLongitude: 139.803333; **Identification:** identifiedBy: L.-P. Kolcsár; **Event:** samplingProtocol: reared; eventDate: 30-04-2020 – 01-05-2020 (reared); **Record Level:** institutionCode: CKLP
- **Type status:** Other material. **Occurrence:** recordedBy: T. Nakamura; individualCount: 1; sex: male; lifeStage: adult; **Location:** island: Honshu; country: Japan; stateProvince: Aomori; municipality: Kawaratai; locality: Nishimeya Vi.; **Identification:** identifiedBy: T. Nakamura; **Event:** samplingProtocol: Malaise trap; eventDate: 2010-06-15 – 2010-08-03; **Record Level:** institutionCode: SCHU
- **Type status:** Other material. **Occurrence:** recordedBy: T. Nakamura; individualCount: 1; sex: male; lifeStage: adult; **Location:** island: Honshu; country: Japan; stateProvince: Aomori; municipality: Hirosaki City; locality: Inekarizawa; **Identification:** identifiedBy: T. Nakamura; **Event:** eventDate: 08/01/2013; **Record Level:** institutionCode: SCHU
- **Type status:** Other material. **Occurrence:** recordedBy: T. Nakamura; individualCount: 7; sex: 6 males, 1 female; lifeStage: adult; **Location:** island: Honshu; country: Japan; stateProvince: Tochigi; municipality: Motegi Town; locality: Ayuta; **Identification:** identifiedBy: T. Nakamura; **Event:** eventDate: 08/09/2005; **Record Level:** institutionCode: SCHU
- **Type status:** Other material. **Occurrence:** recordedBy: K. Sato; individualCount: 1; sex: female; lifeStage: adult; **Location:** island: Honshu; country: Japan; stateProvince: Tochigi; municipality: Utsunomiya City; locality: Tsuruta-numa; **Identification:** identifiedBy: T. Nakamura; **Event:** eventDate: 16/07/1999; **Record Level:** institutionCode: SCHU
- **Type status:** Other material. **Occurrence:** recordedBy: Y. Ohshim; individualCount: 7; sex: 6 males, 1 female; lifeStage: adult; **Location:** island: Honshu; country: Japan; stateProvince: Tochigi; municipality: Utsunomiya City; locality: Tsuruta-numa; **Identification:** identifiedBy: T. Nakamura; **Event:** eventDate: 06/05/2009; **Record Level:** institutionCode: SCHU
- **Type status:** Other material. **Occurrence:** recordedBy: T. Nakamura; individualCount: 2; sex: male; lifeStage: adult; **Location:** island: Honshu; country: Japan; stateProvince: Tochigi; municipality: Utsunomiya City; locality: Tsuruta-numa; **Identification:** identifiedBy: T. Nakamura; **Event:** eventDate: 20/07/2011; **Record Level:** institutionCode: SCHU
- **Type status:** Other material. **Occurrence:** recordedBy: I. Waki; individualCount: 2; sex: 1 male, 1 female; lifeStage: adult; **Location:** island: Honshu; country: Japan; stateProvince: Kanagawa; municipality: Yokohama City; locality: Midori-ku; **Identification:** identifiedBy: T. Nakamura; **Event:** eventDate: 08/11/2002; **Record Level:** institutionCode: SCHU
- **Type status:** Other material. **Occurrence:** recordedBy: D. Kato; individualCount: 1; sex: female; lifeStage: adult; **Location:** island: Honshu; country: Japan; stateProvince: Niigata; municipality: Tokamachi-shi; locality: Matsunoyama-Arahama; verbatimElevation: 310 m; decimalLatitude: 37.09791; decimalLongitude: 138.615166; **Identification:** identifiedBy: D. Kato; **Event:** eventDate: 29/06/2020; **Record Level:** institutionCode: EMMNS
- **Type status:** Other material. **Occurrence:** recordedBy: D. Kato; individualCount: 1; sex: male; lifeStage: adult; **Location:** island: Honshu; country: Japan; stateProvince: Niigata; municipality: Tokamachi-shi; locality: Matsunoyama-Arahama; verbatimElevation: 310 m; decimalLatitude: 37.09791; decimalLongitude: 138.615166; **Identification:** identifiedBy: D. Kato; **Event:** eventDate: 22/06/2020; **Record Level:** institutionCode: EMMNS
- **Type status:** Other material. **Occurrence:** recordedBy: T. Nakamura; individualCount: 2; sex: 1 male, 1 female; lifeStage: adult; **Location:** island: Honshu; country: Japan; stateProvince: Shiga; municipality: Kutsuki Vil; locality: Kijiyama; **Identification:** identifiedBy: T. Nakamura; **Event:** eventDate: 24/07/2011; **Record Level:** institutionCode: SCHU
- **Type status:** Other material. **Occurrence:** recordedBy: T. Nakamura; individualCount: 1; sex: male; lifeStage: adult; **Location:** island: Honshu; country: Japan; stateProvince: Ishikawa; municipality: Hakusan; locality: Super Rindo; **Identification:** identifiedBy: T. Nakamura; **Event:** eventDate: 23/07/2011; **Record Level:** institutionCode: SCHU
- **Type status:** Other material. **Occurrence:** recordedBy: K. Yoshizawa; individualCount: 1; sex: male; lifeStage: adult; **Location:** island: Shikoku; country: Japan; stateProvince: Kochi; locality: Kuroson River; **Identification:** identifiedBy: T. Nakamura; **Event:** eventDate: 26/071996; **Record Level:** institutionCode: SCHU
- **Type status:** Other material. **Occurrence:** recordedBy: K. Kuroda; individualCount: 1; sex: male; lifeStage: adult; **Location:** island: Shikoku; country: Japan; stateProvince: Ehime; municipality: Matsuyama City; locality: Sugesawa-machi; verbatimElevation: 440 m; **Identification:** identifiedBy: T. Nakamura; **Event:** eventDate: 16/17-08-2015; **Record Level:** institutionCode: SCHU
- **Type status:** Other material. **Occurrence:** recordedBy: K. Kuroda; individualCount: 1; sex: female; lifeStage: adul; **Location:** island: Shikoku; country: Japan; stateProvince: Ehime; municipality: Matsuyama City; locality: Tobe-cho; **Identification:** identifiedBy: T. Nakamura; **Event:** eventDate: 18/07/2015; **Record Level:** institutionCode: SCHU
- **Type status:** Other material. **Occurrence:** recordedBy: K. Kuroda et al.; individualCount: 1; sex: female; lifeStage: adul; **Location:** island: Shikoku; country: Japan; stateProvince: Ehime; municipality: Matsuyama City; locality: Mt. Takanawa; **Identification:** identifiedBy: T. Nakamura; **Event:** eventDate: 11/12-08-2016; **Record Level:** institutionCode: SCHU
- **Type status:** Other material. **Occurrence:** recordedBy: T. Nakamura; individualCount: 2; sex: female; lifeStage: adult; **Location:** island: Kyushu; country: Japan; stateProvince: Miyazaki; municipality: Kobayashi; locality: Inokodani, Suki Vil.; **Identification:** identifiedBy: T. Nakamura; **Event:** eventDate: 27/08/2003; **Record Level:** institutionCode: SCHU
- **Type status:** Other material. **Occurrence:** recordedBy: S. Kamitani; individualCount: 1; sex: female; lifeStage: adul; **Location:** island: Kyushu; country: Japan; stateProvince: Kagoshima; locality: Cape Satamisaki; **Identification:** identifiedBy: T. Nakamura; **Event:** eventDate: 05/08/1993; **Record Level:** institutionCode: SCHU
- **Type status:** Other material. **Occurrence:** recordedBy: T. Nakamura; individualCount: 2; sex: 1 male, 1 female; lifeStage: adult; **Location:** island: Kyushu; country: Japan; stateProvince: Oita; municipality: Kohbaru; locality: Foot of Mt. Sobo; **Identification:** identifiedBy: T. Nakamura; **Event:** eventDate: 15/08/1996; **Record Level:** institutionCode: SCHU
- **Type status:** Other material. **Occurrence:** recordedBy: T. Nakamura; individualCount: 1; sex: male; lifeStage: adult; **Location:** island: Kyushu; country: Japan; stateProvince: Miyazaki; municipality: Kobayashi; locality: Inokodani, Suki Vil.; **Identification:** identifiedBy: T. Nakamura; **Event:** eventDate: 24/05/2004; **Record Level:** institutionCode: SCHU
- **Type status:** Other material. **Occurrence:** recordedBy: M. Sueyoshi; individualCount: 1; sex: male; lifeStage: adult; **Location:** island: Kyushu; country: Japan; stateProvince: Kagoshima; municipality: Yamagata Town; locality: Unagi; **Identification:** identifiedBy: T. Nakamura; **Event:** eventDate: 17/05/1996; **Record Level:** institutionCode: SCHU
- **Type status:** Other material. **Occurrence:** recordedBy: T. Nakamura; individualCount: 1; sex: male; lifeStage: adult; **Location:** island: Kyushu; country: Japan; stateProvince: Fukuoka; locality: Mt. Sefuri to Chikushi-yabakei; **Identification:** identifiedBy: T. Nakamura; **Event:** eventDate: 15/06/1996; **Record Level:** institutionCode: SCHU
- **Type status:** Other material. **Occurrence:** recordedBy: T. Nakamura; individualCount: 4; sex: larvae; lifeStage: larva; **Location:** island: Honshu; country: Japan; stateProvince: Aomori; municipality: Hirosaki City; locality: Inekari Zawa; **Identification:** identifiedBy: T. Nakamura; **Event:** eventDate: 19/06/2011; **Record Level:** institutionCode: SCHU
- **Type status:** Other material. **Occurrence:** recordedBy: S. Matsuno; individualCount: 1; sex: larva; lifeStage: larva; **Location:** island: Honshu; country: Japan; stateProvince: Wakayama; municipality: Kainan City; locality: Ebitani; **Identification:** identifiedBy: T. Nakamura; **Event:** eventDate: 18/02/2016; **Record Level:** institutionCode: SCHU
- **Type status:** Other material. **Occurrence:** recordedBy: L.-P. Kolcsár; individualCount: 2; sex: larvae; lifeStage: larva; **Location:** island: Honshu; country: Japan; stateProvince: Yamagata; municipality: Oguni; locality: Arakawa River Valley; verbatimElevation: 340 m; decimalLatitude: 38.192667; decimalLongitude: 139.803333; **Identification:** identifiedBy: L.-P. Kolcsár; **Event:** eventDate: 22/12/2019; **Record Level:** institutionCode: CKLP
- **Type status:** Other material. **Occurrence:** recordedBy: L.-P. Kolcsár; individualCount: 1; sex: larvae; lifeStage: larva; **Location:** island: Shikoku; country: Japan; stateProvince: Kochi; municipality: Tosa; locality: Higashiishihara; verbatimElevation: 705 m; decimalLatitude: 33.695667; decimalLongitude: 133.441833; **Identification:** identifiedBy: L.-P. Kolcsár; **Event:** eventDate: 04/04/2020; **Record Level:** institutionCode: CKLP
- **Type status:** Other material. **Occurrence:** recordedBy: T. Nakamura; individualCount: 1; sex: exuviae; lifeStage: pupa; **Location:** island: Honshu; country: Japan; stateProvince: Aomori; municipality: Hirosaki City; locality: Inekari Zawa; **Identification:** identifiedBy: T. Nakamura; **Event:** eventDate: 19/06/2011; **Record Level:** institutionCode: SCHU
- **Type status:** Other material. **Occurrence:** recordedBy: L.-P. Kolcsár; individualCount: 4; sex: exuviae; lifeStage: pupa; **Location:** island: Honshu; country: Japan; stateProvince: Yamagata; municipality: Oguni; locality: Arakawa River Valley; verbatimElevation: 340 m; decimalLatitude: 38.192667; decimalLongitude: 139.803333; **Identification:** identifiedBy: L.-P. Kolcsár; **Event:** samplingProtocol: reared; eventDate: 30-04-2020 – 01-05-2020 (reared); **Record Level:** institutionCode: CKLP

#### Description

**Descriptions of imago**

**Measurements.** Body length: male 25–36 mm; female: 28–42 mm. Wing length: male 32–43 mm; female 32–42 mm.

**Figure 2.**
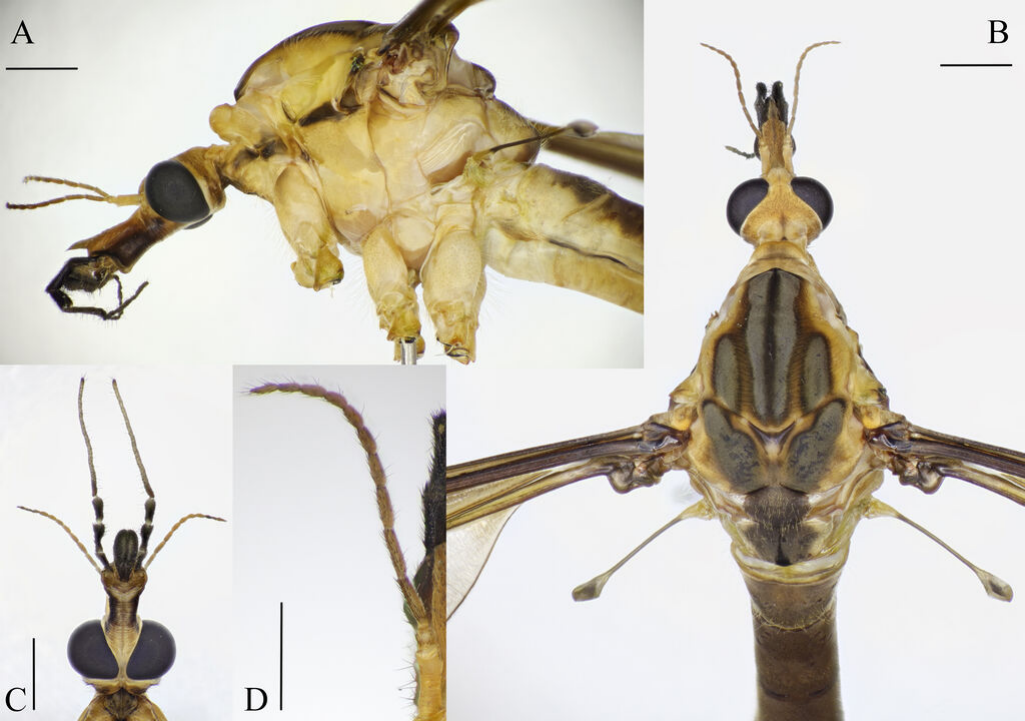
Habitus of *Holorusia mikado* (Westwood, 1876). **A.** Male head and thorax, lateral view; **B.** Male head and thorax, dorsal view; **C.** Female head, ventral view; **D.** Male antenna, dorsal view. Scale bars: 1 mm (A–C); 0.5 mm (D).

**Head.** Yellowish-brown; ventral side of rostrum dark brown, with short brown setae (Fig. 2A–C). Rostrum as long as rest of head; nasus present. Labellum black. Palpus five-segmented; longer than antenna. Last palpomere paler and longer than rest of palpus (Fig. 2A and B). Antenna shorter than head; scape yellowish-brown, about 2.5 times as long as it is wide; pedicel oval and slightly darker than scape; flagellum 10–12, segmented, light brown to brown; flagellomeres cylindrical, basal flagellomeres longer, distal flagellomeres shorter; verticels shorter than diameter of flagellomeres (Fig. 2D).

**Thorax.** Yellowish-brown, with brownish and grey markings. Presutural area of scutum with three grey, longitudinal stripes; median stripe with a dark brown line in the middle; stripes surrounded with brown as in Fig. 2B. V-shaped transverse suture with triangular, dark patch with whitish peaks; surrounded by yellowish-brown area (Fig. 2B). Postsutural area of scutum with two grey stripes. Scutellum grey, with whitish corners. Mediotergite greyish-brown, with longitudinal dark brown line in the middle; laterally yellowish-brown as in Fig. 2B. Lateral part of thorax whitish-yellow to yellowish-brown in living specimens and yellowish-brown in preserved specimens; a dark brown line from the cervical sclerite to base of wing as in Fig. 2A. Base of halter yellowish; steam and knob dark brown (Fig. 2A and B).

**Figure 3.**
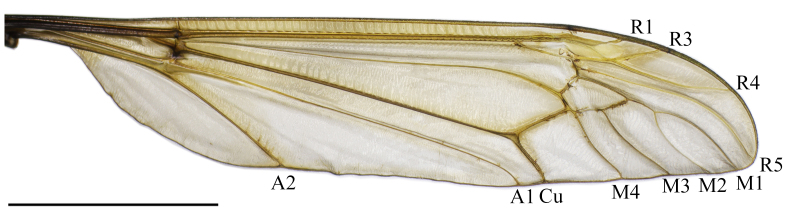
Wing of *Holorusia mikado* (Westwood, 1876). Scale bar: 1 cm.

**Wing.** Tinged with brown. Base of wing dark brown; Sc and R black proximal to crossvein h, remaining veins light brown to brown. Stigma inconspicuous. Calypter bare and well developed. Venation as in Fig. 3.

**Figure 4.**
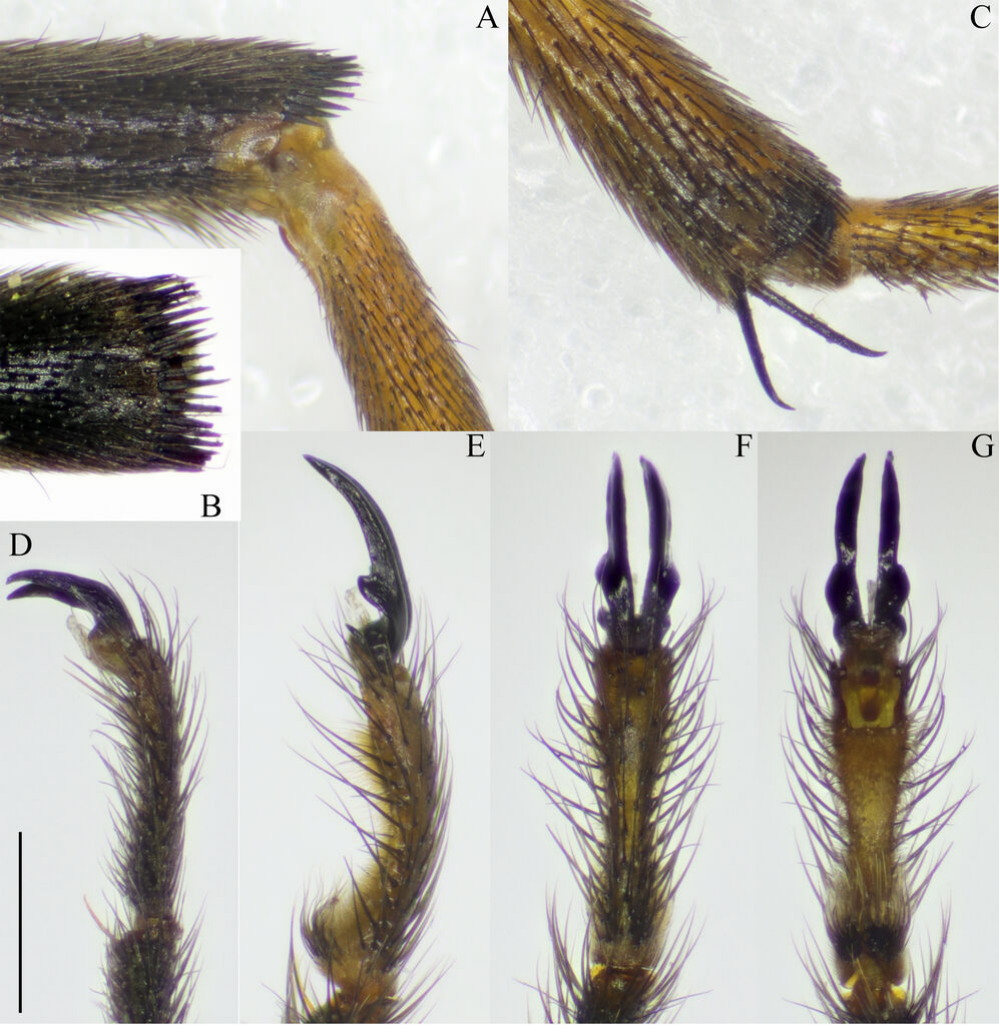
Leg parts of *Holorusia mikado* (Westwood, 1876). **A.** Tip of femur with comb of setae; **B.** Tip of the femur with comb of setae; **C.** Tip of tibia; **D.** Female fifth tarsomere, lateral view; **E.** Male fifth tarsomere, lateral view; **F.** Male fifth tarsomere, dorsal view; **G.** Male fifth tarsomere, ventral view. Scale bar: 0.5 mm (D–G).

**Legs.** Light brown to brown. Tips of femur and tibia each with a narrow black ring. Tip of femur with comb of dark, strong setae (Fig. 4A and B). Tip of tibia with less prominent comb of setae. Tibial spurs shorter than width of tibia; tip of spur curved (Fig. 4C); formula: 1, 2, 2. Last tarsomere slightly curved ventrally in males (Fig. 4E), almost straight in females (Fig. 4D). Base of male last tarsomere with hairy lobe (Fig. 4E and G). Female tarsal claw simple (Fig. 4D); male tarsal claw with small sharp tooth at its base and a blunt tooth at ¼ of length of claw (Fig. 4E and G).

**Figure 5.**
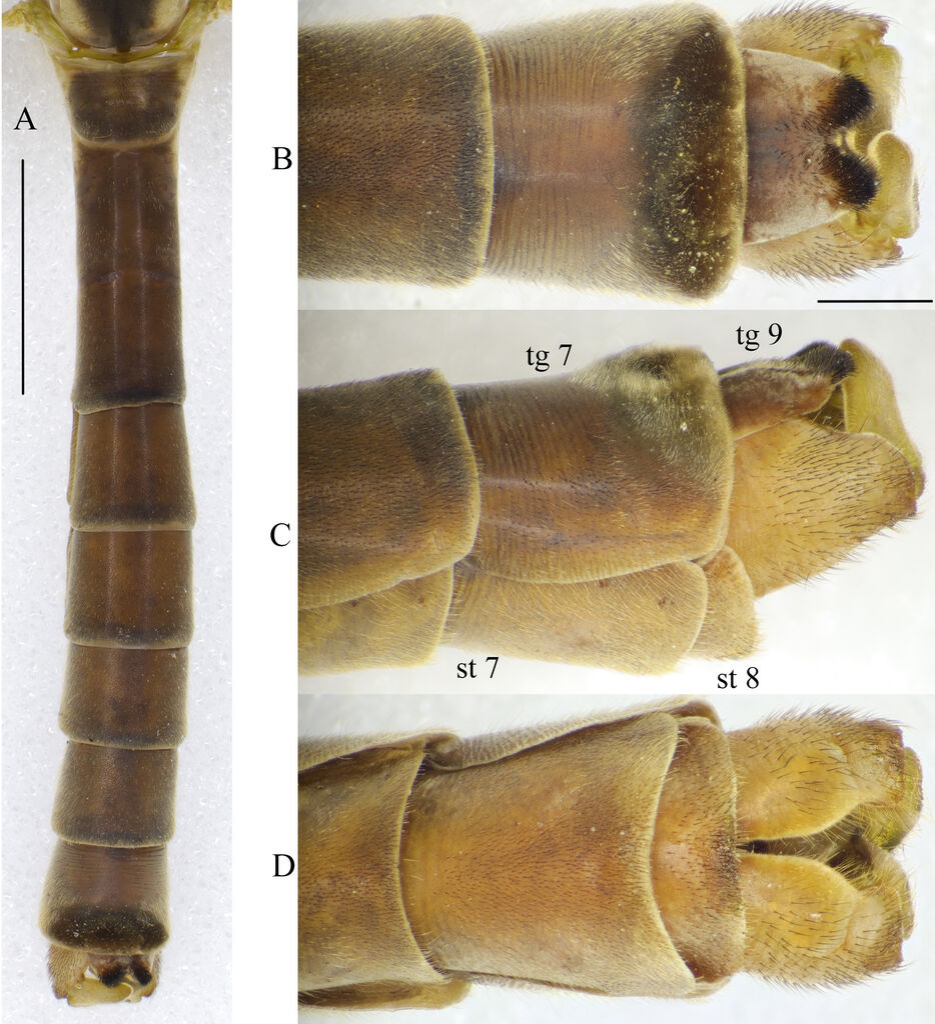
Abdomen of male *Holorusia mikado* (Westwood, 1876). **A.** Abdomen, dorsal view; **B.** Male terminalia, dorsal view; **C.** Male terminalia, lateral view; **D.** Male terminalia, ventral view. Scale bars: 5 mm (A); 1 mm (B–D).

**Figure 6.**
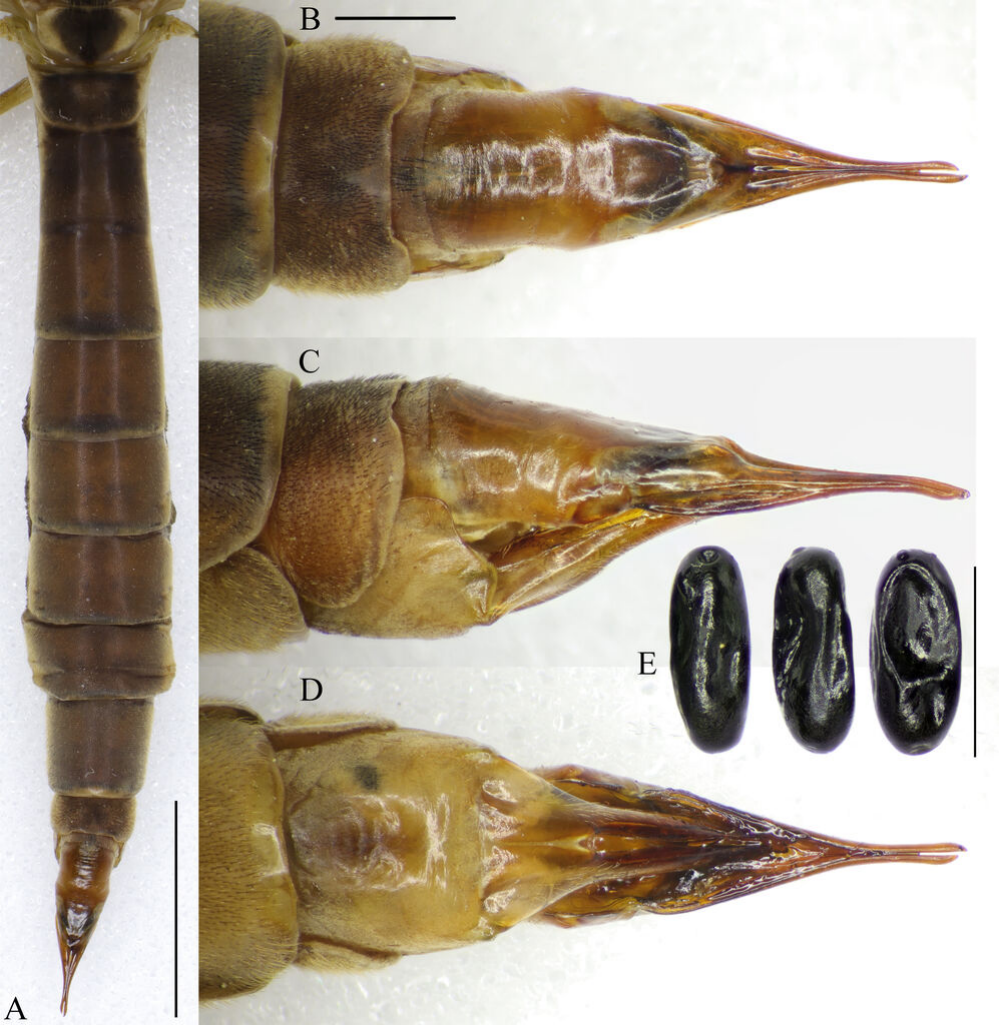
Abdomen of female *Holorusia mikado* (Westwood, 1876). **A.** Abdomen, dorsal view; **B.** Female terminalia, dorsal view; **C.** Female terminalia, lateral view; **D.** Female terminalia, ventral view; **E.** Unfertilised eggs. Scale bars: 5 mm (A); 1 mm (B–D), 1 mm (E).

**Abdomen.** Dark brown on both sexes (Figs 5, 6). Tergites slightly darker than sternites. Posterior margin of tergites slightly darker in both sexes. Anterior part of male tergite 7 bare, with transverse lines; posterior part covered by very dense, dark brown, brush of hairs (Fig. 5A). Male tergite 8, short, bare, telescopic and retracted under tergite 7, not visible externally.

**Figure 7.**
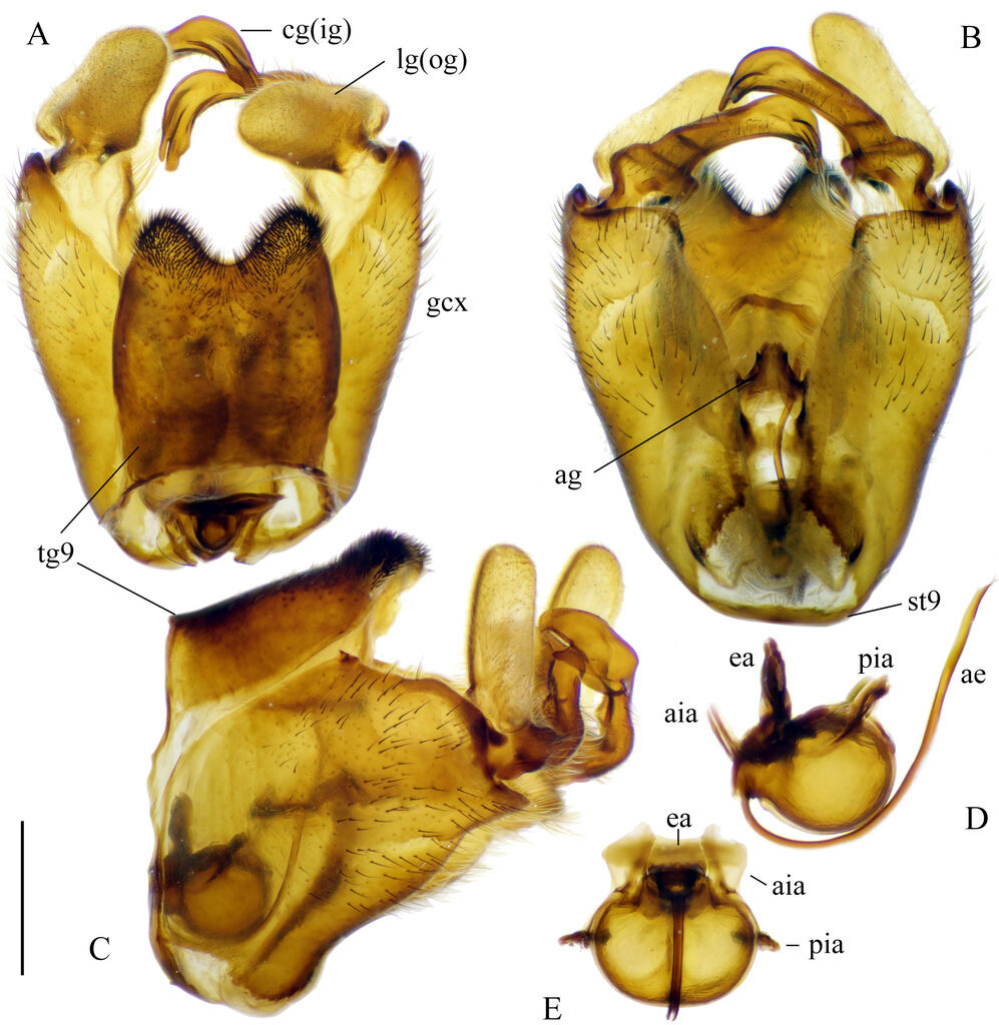
Male genitalia of *Holorusia mikado* (Westwood, 1876). **A.** Dorsal view; **B.** Ventral view; **C.** Lateral view; **D.** Sperm pump and aedeagus, lateral view; **E.** Sperm pump, anterior view. Abbreviations: **ae** – aedeagus, **ag** – aedeagal guide, **aia** – anterior immovable apodeme, **ea** – ejaculatory apodeme, **pia** – posterior immovable apodeme, **gcx** – gonocoxite, **cg(ig)** – clasper of gonostylus (inner gonostylus, **lg(og)** – lobe of gonostylus (outer gonostylus), **tg9** – tergite 9, **st** – sternite 9. Scale bar: 1 mm (A–C).

**Figure 8.**
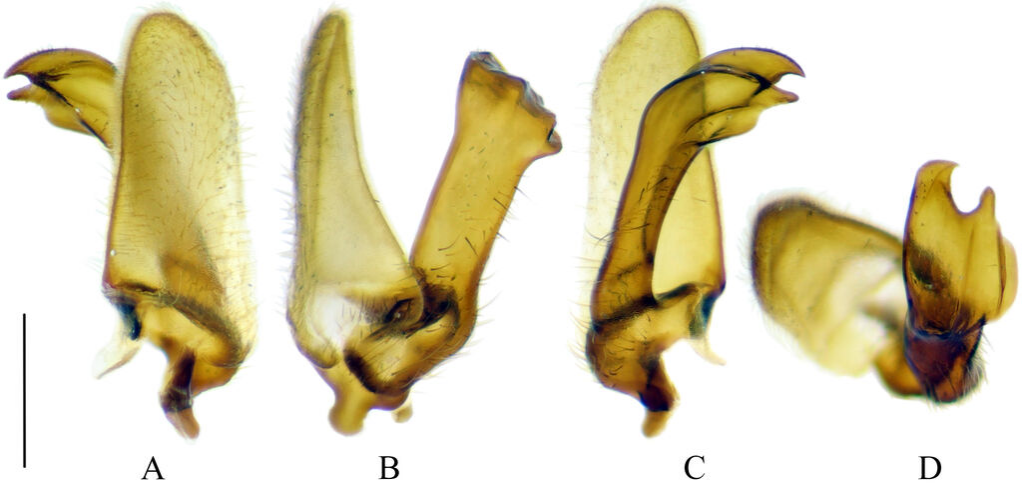
Gonostylus of *Holorusia mikado* (Westwood, 1876). **A.** Gonostylus, outer lateral view; **B.** Gonostylus, caudal (ventral) view; **C.** Gonostylus, inner lateral view; **D.** Tip of gonostylus. Scale bar: 0.5 mm.

**Male terminalia.** Yellowish-brown to brown (Fig. 5B–D). Tergite 9 brown, relatively small, shorter than gonocoxite. Posterior margin of tergite 9 covered by dense dark setae; with deep notch in middle (Fig. 7A). Sternite 9 reduced to narrow band; fused with gonocoxites (Fig. 7B). Gonocoxite as long as wide in lateral view; bulbous outgrowths on anterior-ventral side (Fig. 7C). Gonocoxites not fused, connected by membranous area (Fig. 7B). Gonostylus relatively simple. Lobe of gonostylus (outer gonostylus) fleshy, 2.2 times longer than wide, covered by fine pale hairs (Fig. 8). Clasper of gonostylus (inner gonostylus) sclerotised, curved dorsally and widening apically; fist-like, with two acute tips (Fig. 8). Aedeagus complex typical as in Tipulinae. Sperm pump as wide as long (Fig. 7D and E), anterior and posterior immovable apodemes equal in length; ejaculator apodeme 1.3–1.5 times longer than immovable apodemes (Fig. 7D). Aedeagus 2–2.2 times longer than length of sperm pump; tip with two filaments (Fig. 7D). Aedeagal guide triangular as in Fig. 7B.

**Figure 9.**
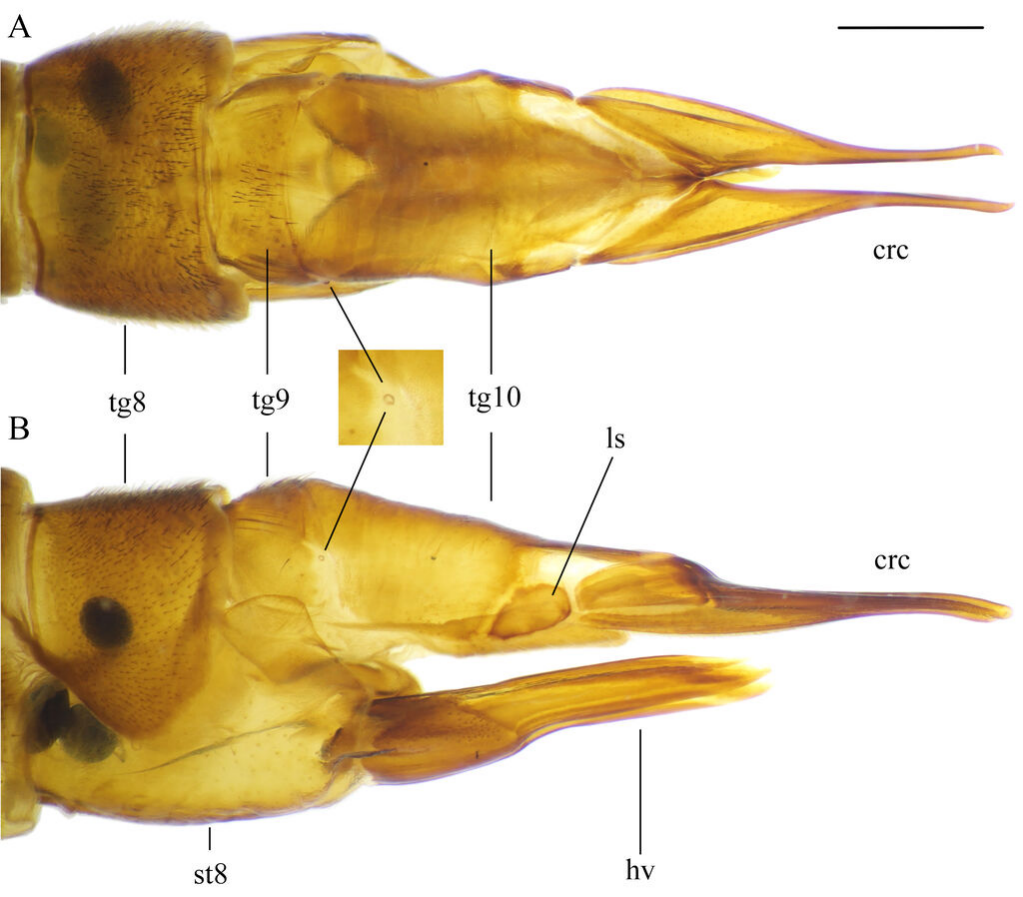
Female terminalia of *Holorusia mikado* (Westwood, 1876). **A.** Dorsal view; **B.** Lateral view. Abbreviations: **crc** – cercus, **hv** – hypogynial valve, **ls** – lateral sclerite, **tg** – tergite, **st** – sternite. Scale bar: 1 mm.

**Figure 10.**
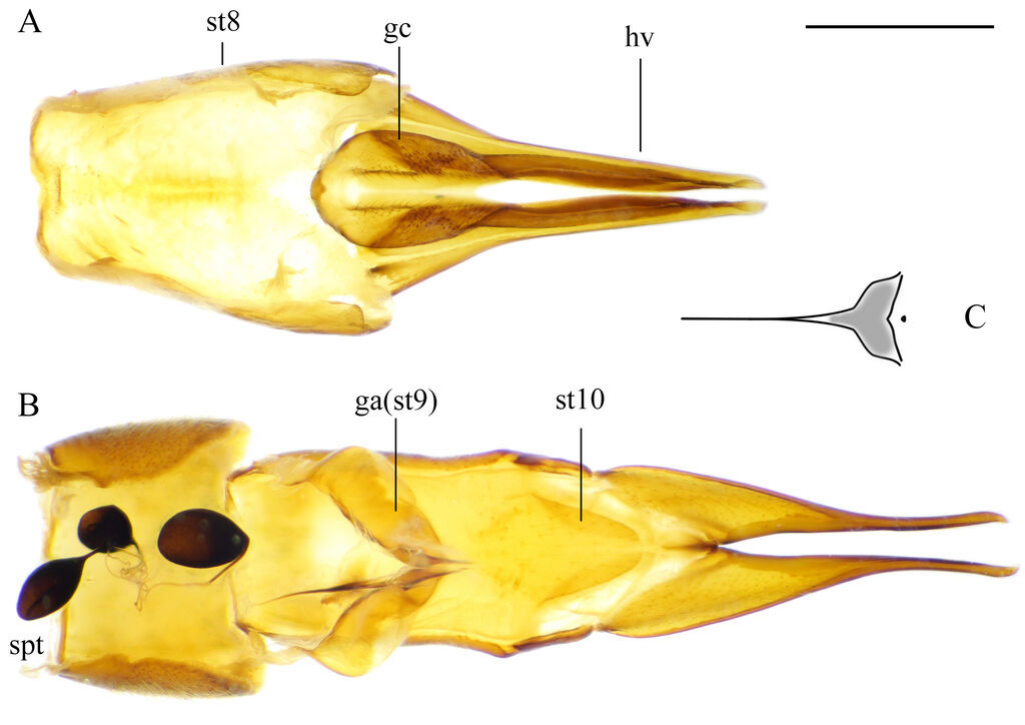
Female terminalia of *Holorusia mikado* (Westwood, 1876). **A.** Sternite 8 and hypogynial valves, dorsal view; **B.** ovipositor after sternite 8 and hypogynial valves removed, ventral view; **C.** Genital fork. Abbreviations: **gc** – genital chamber, **ga(st9)** – genital apodema/sternite 9, **hv** – hypogynial valve, **spt** – spermathecae, **st** – sternite. Scale bar: 1 mm.

**Female terminalia.** Light brown to brown Fig. 6. Tergite 8 not fused with tergite 9; tergites 9 and 10 fused. Tergite 8 with black setae; V-shaped bare area in the middle of posterior margin (Fig. 6B and Fig. 9A). Tergite 9 with several black setae at middle (Fig. 6B and Fig. 9A). Tergite 9 half as long as tergite 10; covered anteriorly by tergite 8 and ventrally by sternite 8. Small pit between tergites 9 and 10 on lateral side (Fig. 9A and B). Lateral sclerite (lobe) of tergite 10 darker than rest of tergite 10. Cercus fused with tergite 10. Tergite 10 and cercus bare and polished (Fig. 6B and C). Cercus as long as hypogynial valve, very narrow and slightly curved ventrally (Fig. 9B). Sternite 8 and hypogynial valve with pale setae. Hypogynial valve 1.2–1.3 times longer than sternite 8. Genital fork triangular; widening toward caudal end (Fig. 10C). Vaginal apodeme as in Fig. 10B. Genital chamber with a few short setae (Fig. 10A). Sternite 10 slightly longer than wide; rounded proximally. Three spermatheca black, differing in shape, from elongated to more rounded with straight or curved ducts (Fig. 10B).

**Egg.** A female laid, deformed, unfertilised eggs (not copulated with male) 5 days after it emerged from pupa. Shape of fertilised eggs may differ than that which is described below.

Eggs shiny black, 1.1–1.2 mm long, surface without granulation; micropylar opening (micropyle) at subapical protrusion or in small pit (Fig. 6E).

**Description of last instar larva**

**Measurements.** Length 45–55 mm (n = 11), width 6–7 mm (n = 11).

**Figure 11.**
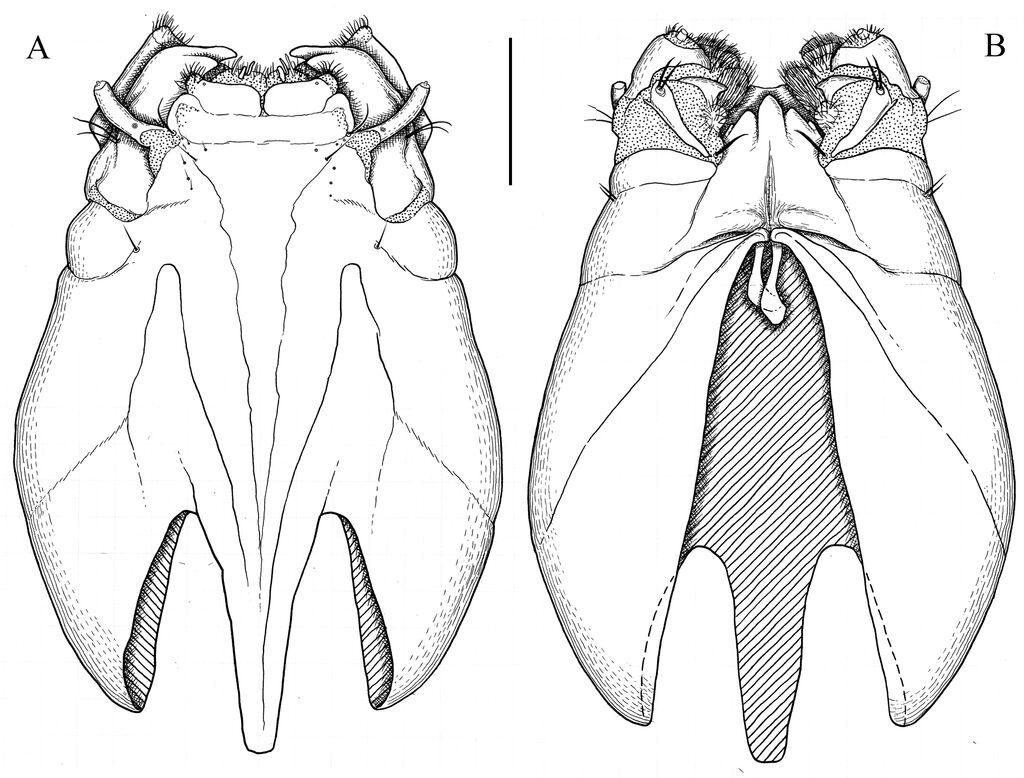
Head capsule of last instar larva of *Holorusia mikado* (Westwood, 1876). **A.** dorsal view; **B.** ventral view. Scale bar: 1 mm.

**Head capsule.** Length 4.5–5 mm (n = 4), width 2.5–2.8 mm (n = 4); oval in shape, massive tipulid type (Fig. 11). Incisions reach almost 1/3 of length of head. Internolateralia and externolateralia more sclerotised anteriorly and less sclerotised, subhyaline posteriorly. Frontoclypeal area and interolateralia separated by frontal suture. Frontoclypeal area narrowing posteriorly. Dorsal endocarina present, with paired ridges. Externolateraliae widely separated on the ventral side, incision elongated U or V-shaped.

**Figure 12.**
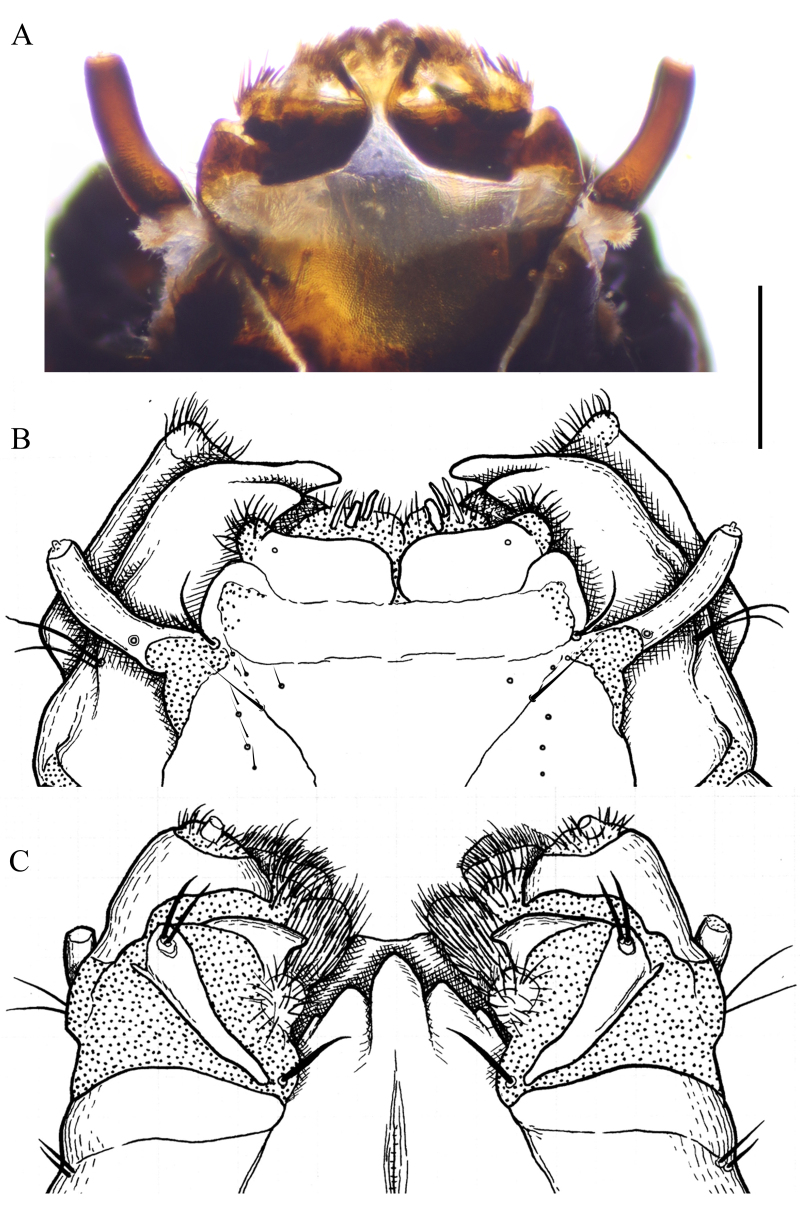
Anterior parts of head capsule of the last instar larva of *Holorusia mikado* (Westwood, 1876). **A.** labrum, frontoclypeus and antennae after maxillae and mandibles removed, dorsal view; **B.** anterior part of head capsule, dorsal view; **C.** anterior part of head capsule, ventral view. Scale bar: 0.5 mm.

Lateral sclerotised plates of labrum close situated, distance less than 1/6 of plate width (Fig. 12A and B). Each plate with two parts anteriorly. Outer-lateral part with 10-15 spine-like setae and with two small pores, one on ventral, one on dorsal side. Inner-lateral part membranous covered with hairs; one finger-like black sensory seta on the inner corner; two longer, hyaline, flattened setae laterally to black seta; additional two-three poorly visible papillae amongst hairs. Membranous part between sclerotised plates of labrum, directed ventrally, medially with a notch, covered with stronger hairs (Fig. 12A) and with two papillae, visible only in apical view. Membranous articulation point of antenna with pale, short, dense hairs. Antenna cylindrical, 3–3.5 times longer than wide; slightly curved inwards; sensory pit close to base of antenna (Fig. 12A, B).

Anterior part of clypeus weakly sclerotised. Eight setae around base of antenna; three equal in size, short setae, along outer side of frontal suture; five setae along inner side of frontal suture as: one long, pale seta near base of antenna, two small and one longer setae along frontal suture and one short seta near inner-laterally (Fig. 12B).

**Figure 13.**
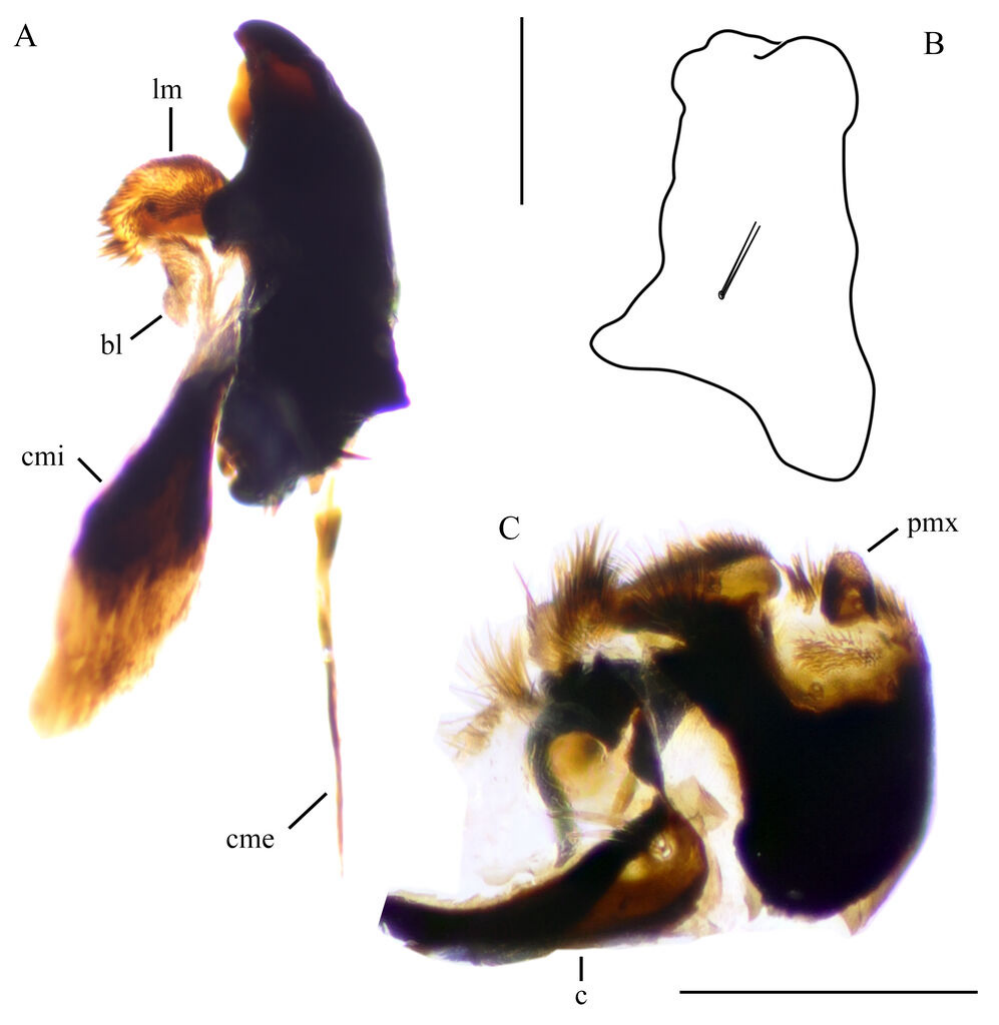
Mouthparts of head capsule of last instar larva of *Holorusia mikado* (Westwood, 1876). **A.** Mandible, ventral view; **B.** outline of mandible, outer lateral view; **C.** Maxilla, ventral view. Not to scale. Abbreviations: **bl** – basal lobe of lacinia mobilis, **c** – cardo **cmi** – craniomandibularis internus, **cme** – craniomandibularis externus, **lm** – lacinia mobilis, **pmx** – maxillary palp. Scale bars: 0.5 mm (A and B); 0.5 mm (C).

Mandible massive, rectangle in lateral view, with two sensory setae at base on lateral side (Fig. 13B). Two blunt teeth on inner side; lacinia mobilis almost as wide as mandible, covered with short blunt setae at base and on middle; tip with longer setae. Additional membranous lobe present below the lacinia mobilis; partly connected to craniomandibularis internus. Craniomandibularis internus well-developed, as long as mandible, strongly sclerotised, flattened dorso-ventrally. Craniomandibularis externus rod-shaped and as long as mandible (Fig. 13A).

Maxilla well developed; cardo triangular, slightly curved, with two pale, long setae near to distal end. Long seta with short base at membranous part of maxilla near the joinpoint of maxilla and hypostomal plate (Fig. 12C). Remaining part of maxilla formed by three sclerites. Small triangular sclerite anteriorly to cardo. Inner and outer sclerites with several membranous areas and lobes. Inner sclerite with an apical lobe, covered with long, dorsally curved seate; an inner lateral lobe, covered with long inner-dorsally curved setae; a membranous area on dorsal side near base, with apically directed setae. Outer sclerite with membranous palpifer, with tuft of hairs at the apex, four sensory pits around palp as: two on ventral side next to sclerotised area, one outer-laterally to palp, one inner-laterally to palp; palp short, with a sensory pit at base, tip with membranous sensory ring-like structure; membranous lobe at tip of maxilla densely covered with strong dorsally curved setae, two short, unequal and finger-like papillae barely visible (Figs 12, 13).

**Figure 14.**
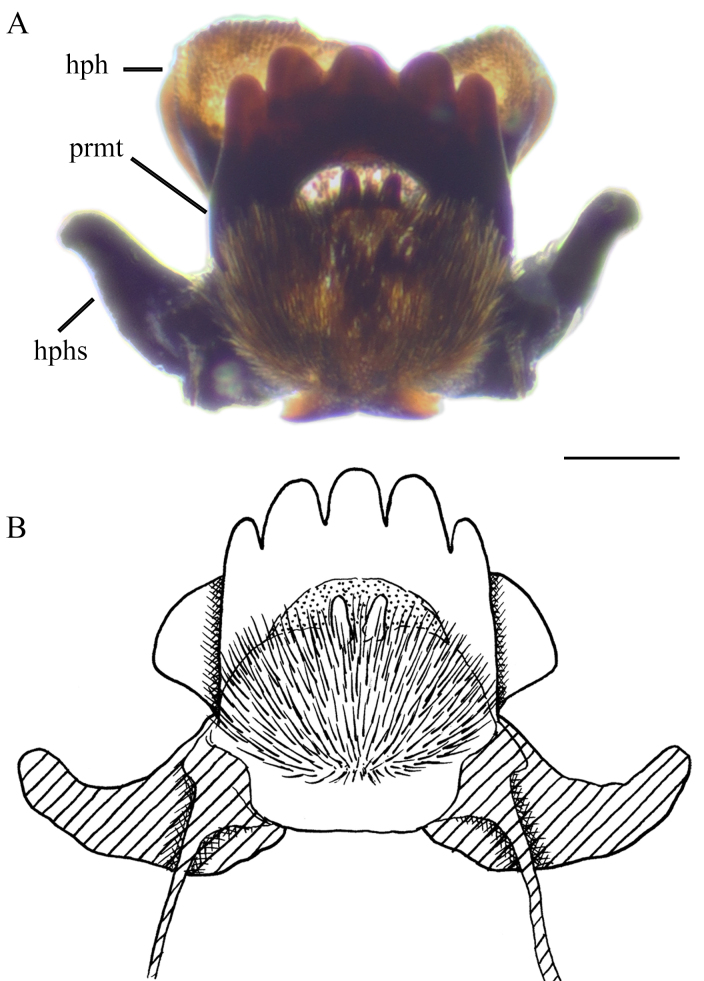
Hypopharynx and prementum of last instar larva of *Holorusia mikado* (Westwood, 1876). Abbreviations: **hph** – hypopharynx, **hphs** – hypopharyngeal suspensoria, **prmt** – prementum. Scale bar 0.2 mm.

Prementum with five blunt teeth; labial area with two papillae on middle, ventral side covered by group of dense hairs originating from posterior premental margin. Hypopharynx membranous, more or less bilobed and covered by short, dense hairs. Hypophayngeal suspensoria (lateral arms of hypopharynx) sclerotised and curved apically (Fig. 14).

**Figure 15.**
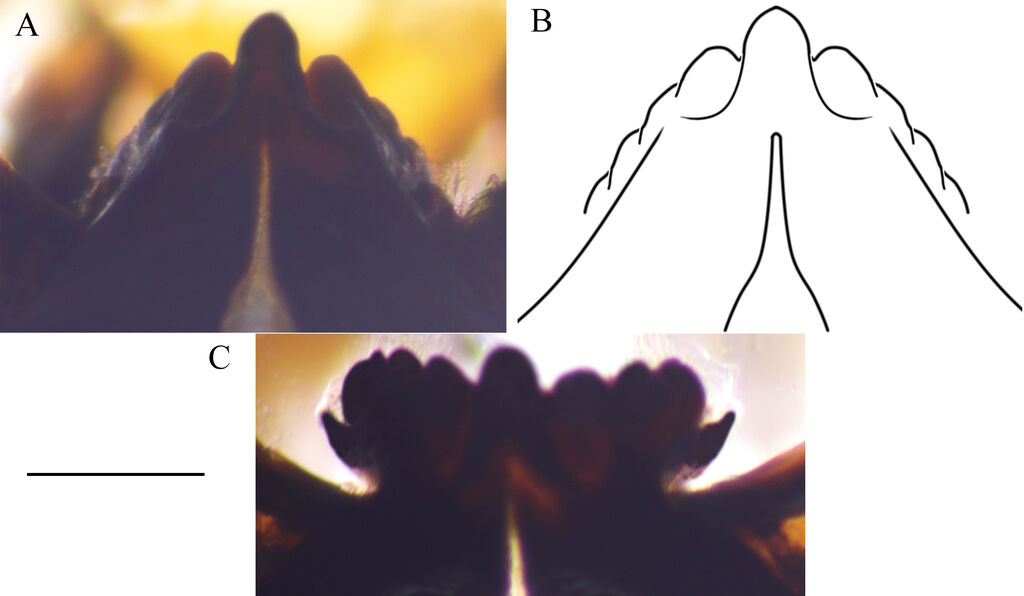
Hypostomium of last instar larva of *Holorusia mikado* (Westwood, 1876). **A**, **B.** Hypostomium plate, ventral view; **C.** Hypostomium plate, apical view after maxilla and mandibles removed. Scale bar 0.5 mm.

Hypostomal plate with 9 teeth; middle tooth most prominent (Fig. 15A and B), outermost tooth poorly developed, covered by membranous part of maxilla, visible only after removing maxilla, in apical view (Fig. 15C).

**Figure 16.**
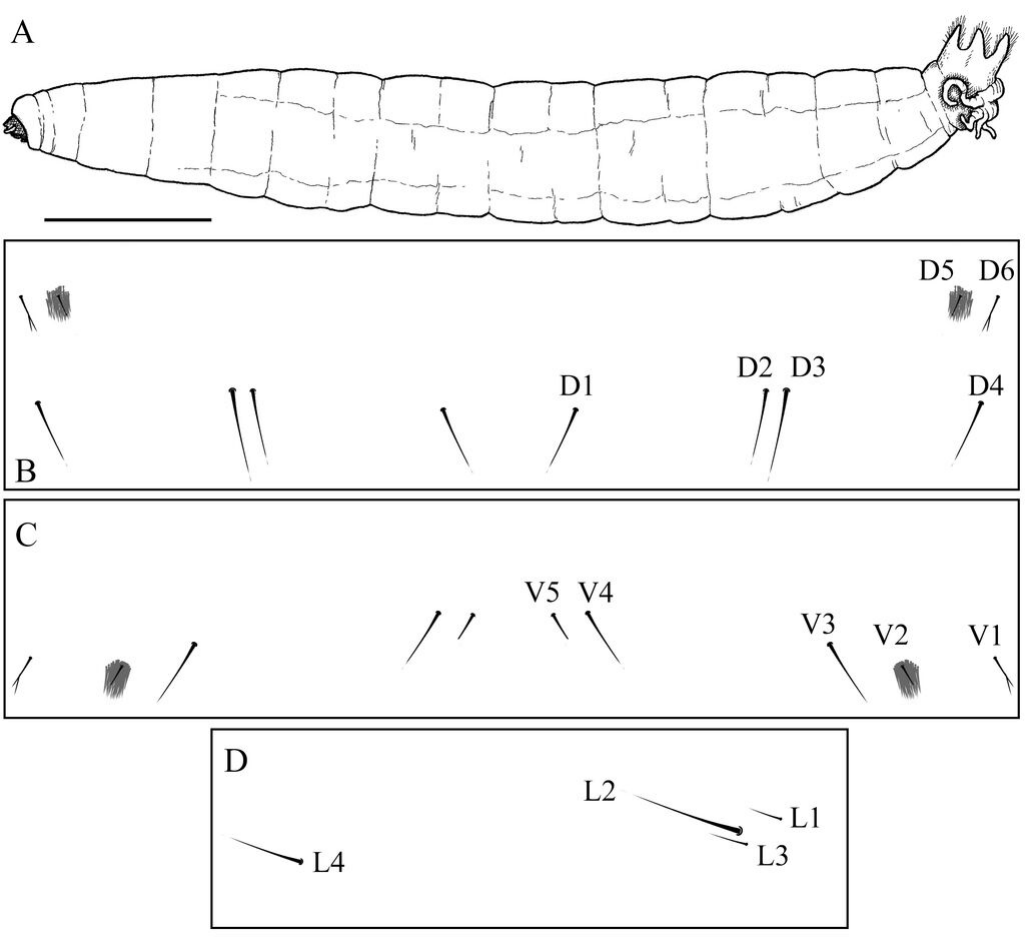
Last instar larva of *Holorusia mikado* (Westwood, 1876). **A.** Lateral view; **B**–**D.** chaetotaxy of abdominal segment II–VII. **B.** Dorsal setae; **C.** Ventral setae; **D.** Pleural (lateral) setae. Scale bar: 1 cm (A).

**Thorax and abdomen.** Living specimen greenish-brown, specimen stored in ethanol blackish-brown. Dorsum covered with micro and macro setae, not forming clear patches. Micro setae less dense on ventrum. Pleural area with dense setae, especially conspicuous in specimens stored in ethanol, due to shrinkage of the pleural area. Chaetotaxy of abdominal segment II–VII as in Fig. 16B–D. Setae D6 and V1 bifid. D5 and V2 with patches of dense setae. L3 closer to L2 than to L1.

**Figure 17.**
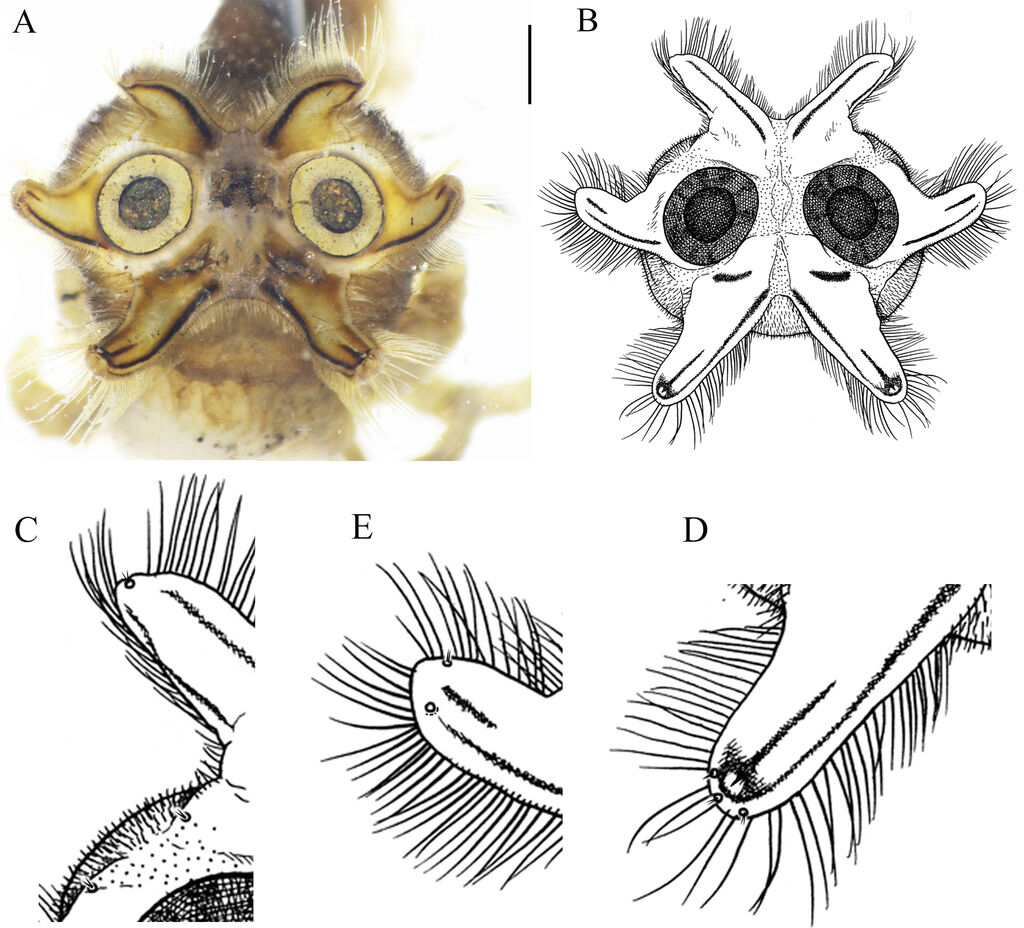
Spiracular disc of last instar larva of *Holorusia mikado* (Westwood, 1876). **A.** photo; **B.** drawing; **C.** dorsal lobe and area between dorsal and lateral lobe; **D.** lateral lobe; **E.** ventral lobe. Scale bar: 1 mm (A and B).

**Anal division and spiracular disc.** Spiracular lobes subequal in length; ventral lobe slightly longer. Lobes capable of closing and hiding spiracles. Spiracles large, more than 1/3 as wide as spiracular disc. Margin of lobe fringed with long pale setae, base of setae black. Dorsal and ventral lobes with dark line along inner margin of lobe; lateral lobe with dark line along ventral margin. Lateral and ventral lobes with short black line, 1/3 of length of lobe. Dorsal lobe with a less noticeable line along the outer margin. Tip of ventral lobe with infuscated area. Sclerotised area ventrally to spiracle narrow line, length half of width of spiracle. Shade of patterns on lobes variable amongst specimens, delicated line on dorsal lobe sometimes difficult to recognise. (Fig. 17A and B.)

Sensory setae are very short, very difficult to distinguish them. It is not clear if the short setae are bifid or two separate setae arising from each alveolus.

Dorsal lobe with alveolus at tip of lobe (Fig. 17A). Lateral lobe with alveolus at tip of lobe, dorsally to short mid-line; a sensatory pit at tip of ventral line (Fig. 17E). Ventral lobe with a black spine-like papilla subapically; lobe pale around the base of papilla; additional three alveoli as: one alveolus apical to black papilla, one-one alveolus lateraly to black papilla (Fig. 17C). Two additional alveoli between base of dorsal and lateral lobes, distance between alveoli around 1/3-1/4 of width of spiracle (Fig. 17A).

**Figure 18.**
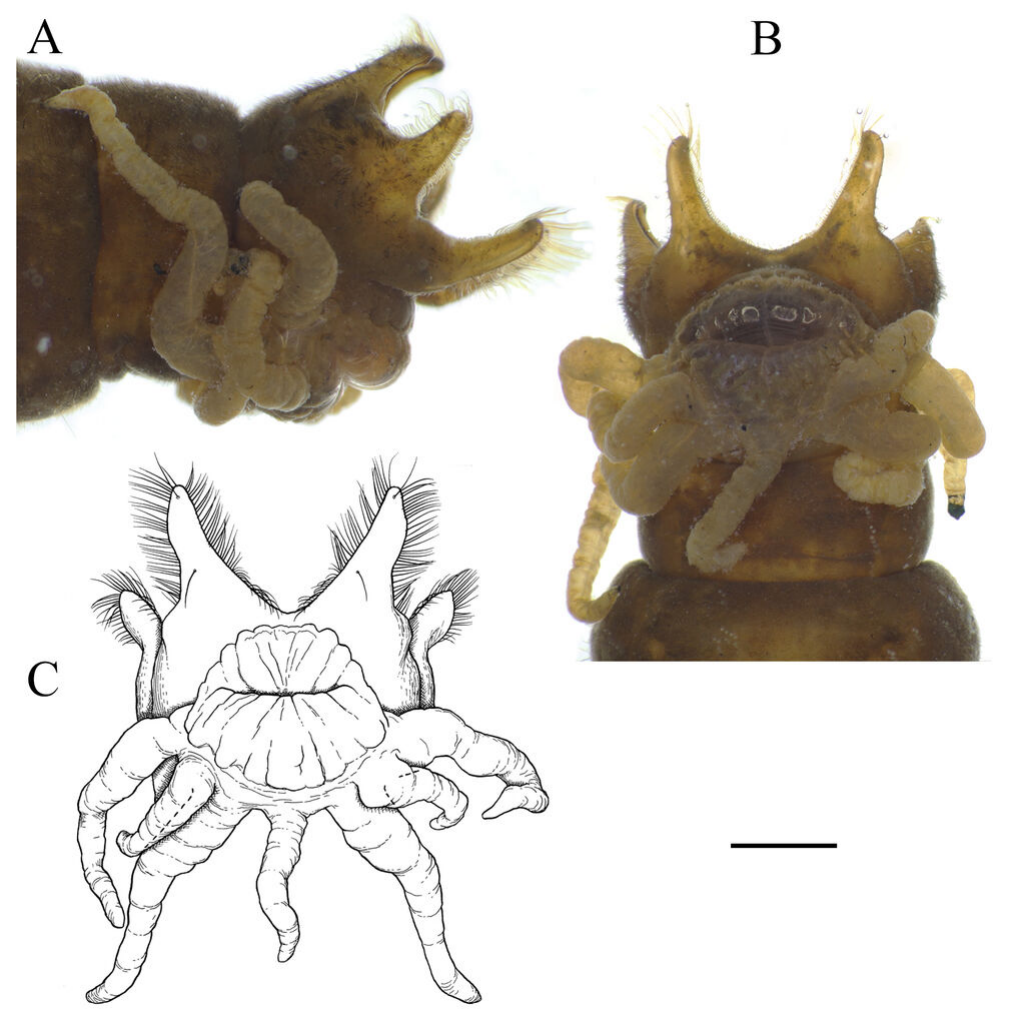
Anal field of last instar larva of *Holorusia mikado* (Westwood, 1876). **A.** photo, lateral view; **B.** photo, ventral view; **C.** drawing, ventral view. Scale bar: 2 mm

Anus surrounded by seven yellowish-brown, fleshy, long anal papillae. Three papillae on lateral side, one unpaired papilla at anterior margin of anus (Fig. 18). Three lateral papillae differing in length, anterior papilla longest. In living specimens, papillae about 1.5 times longer than those in Fig. 18A. Lateral papillae bent dorsally, unpaired directed anteriorly (Fig. 18).

**Description of pupa**

**Measurements**. Length 35–50 mm (n = 4), width 5.5–6.5 mm (n = 4).

**Figure 19.**
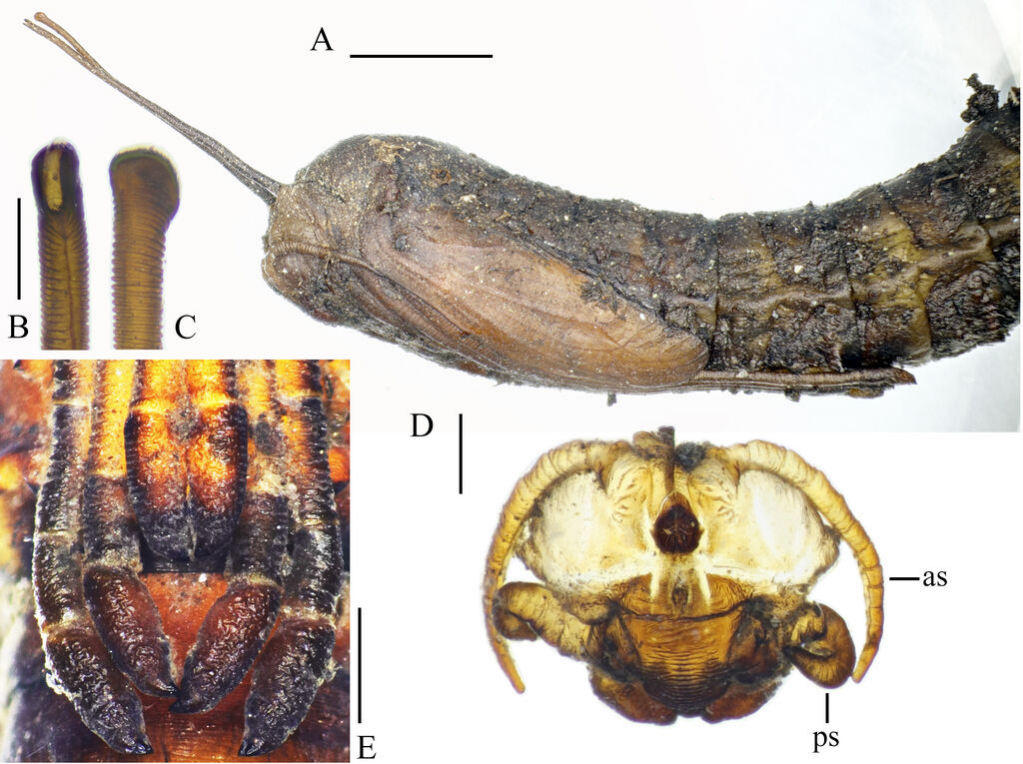
Pupa of *Holorusia mikado* (Westwood, 1876). **A.** Anterior part, lateral view; **B.** Tip of respiratory horn, dorsal view; **C.** Tip of respiratory horn, lateral view; **D.** head sheath. Abbreviations: **as** – antenna sheath, **ps** – palpus sheath. Scale bars: A 5 mm (A); 0.5 mm (B–C); 1 mm (D–E).

General colouration dirty brown. Pupal skin covered with fine particles of substrate (Fig. 19A).

**Head.** Antennal sheath short, as long as head sheath. Labrum sheath large, with transverse wrinkles; labial sheaths separated from each other; palpus sheath recurved (Fig. 19D).

**Thorax.** Respiratory horns 1.75 times longer than width of thorax in lateral view (Fig. 19A). Horns equal in length, ringed, apical end flattened laterally and slightly widened; with large, longitudinal opening (Fig. 19B and C). Horn of pupae in wet environments straight; horn of specimens kept in dry condition curved ventrally. Wing sheath reaches posterior end of second abdominal segment, wing venation distinct (Fig. 19A). Leg sheaths extend just beyond posterior end of third abdominal segment. Fore leg sheath shortest, reaching posterior end of fourth tarsomere of mid-leg; hind leg longest. Last tarsomere extended in both sexes, but more prominent in males (Fig. 19E). Tarsal claw sheath prominent in males (Fig. 19E), less in females.

**Figure 20.**
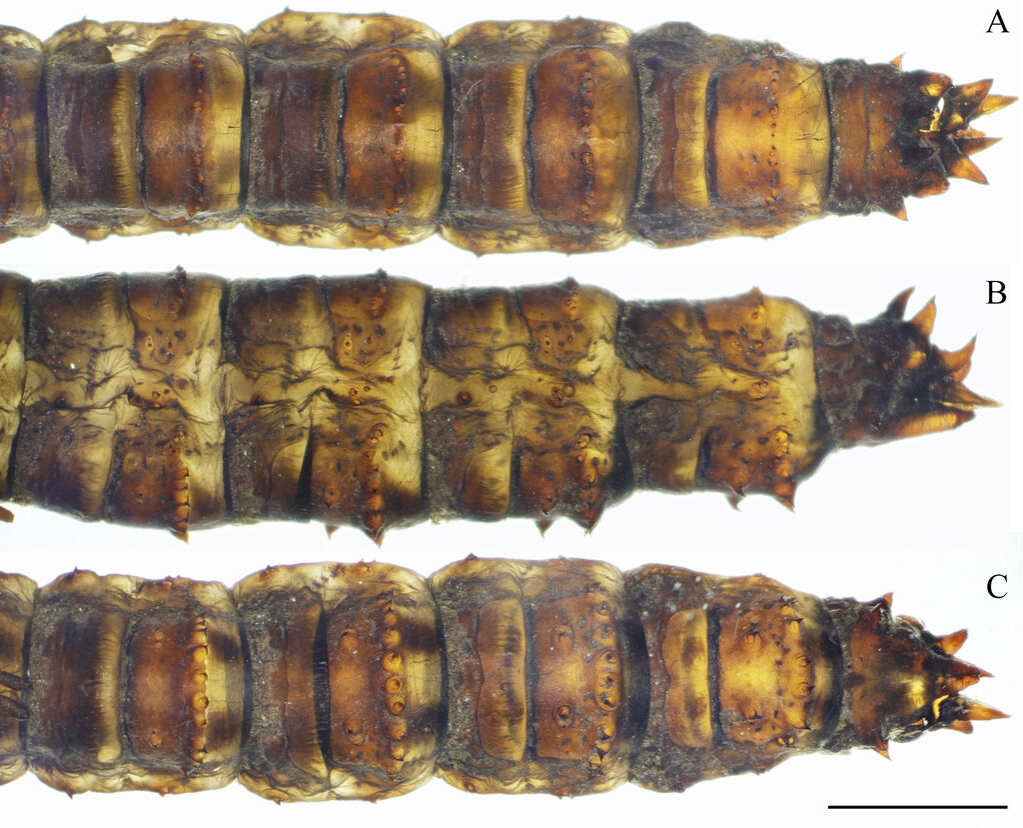
Female abdomen of pupa of *Holorusia mikado* (Westwood, 1876). **A.** dorsal view; **B.** lateral view; **C.** ventral view. Scale bar: 5 mm.

**Abdomen.** Pleurites flattened dorso-ventrally (Fig. 20A). Spines strong, acutely ending; ventral spines longer than dorsal spines and posterior spines longer than anterior spines. Number of dorsal spines on each segment (IV to VII) 18–22; number of ventral spines: 18–20 on segments IV to VI and 14–16 on segment VII. Pleurites with four spines (Fig. 20).

**Figure 21.**
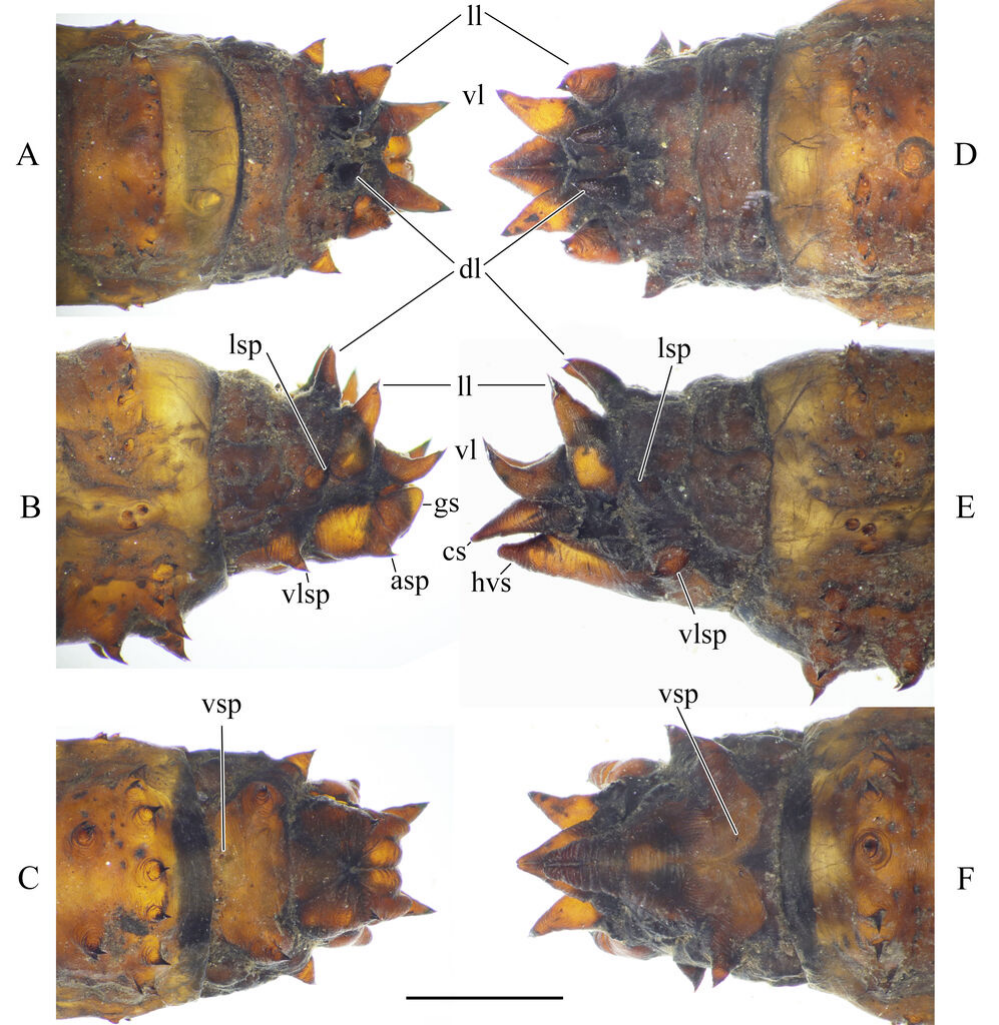
Posterior end of pupa of *Holorusia mikado* (Westwood, 1876). **A**–**C.** male: **A.** dorsal view; **B.** lateral view; **C.** ventral view. **D**–**F.** female: **D.** dorsal view; **E.** lateral view; **F.** ventral view. Abbreviations: **ad** – anterio-dorsal spine, **as** – anal spine, **cs** – cerci sheath, **gs** – genital sheath, **hvs** - hypogynial valva sheath, **ls** – lateral spine, **md** – medio-dorsal spine, **pd** – posterio-dorsal spine, **vls** – ventro-lateral spine, **vs** – ventral spine. Scale bar: 2.5 mm.

**Figure 22.**
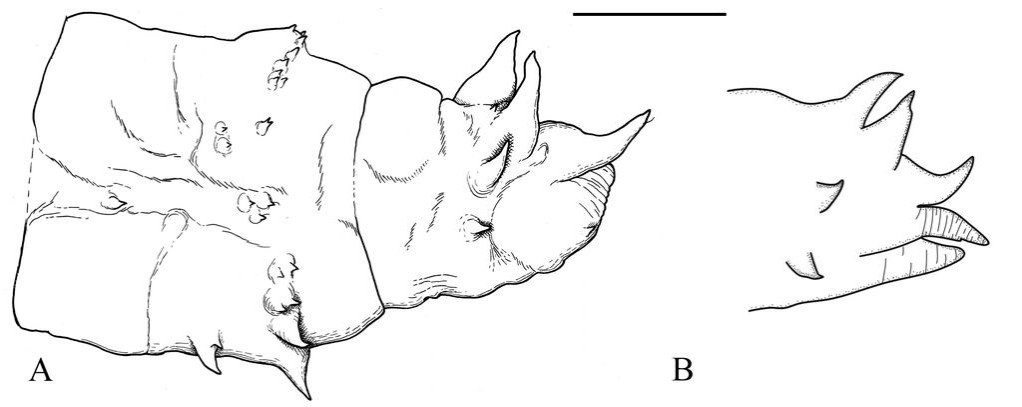
Lateral view of the posterior end of pupa of *Holorusia mikado* (Westwood, 1876). **A.** male; **B.** female. Scale bar: 2.5 mm.

**Male pupa**. Last abdominal segment armed with six dorsal acute spine, equivalent to lobes of larval spiracular field (Fig. 21A, B and Fig. 22A), posterio-dorsal spine curve dorsally (Fig. 21B and Fig. 22A). Genital sheath blunt, with prominent anal spine (Fig. 21B and Fig. 22A). Lateral spine slightly anterior to posterio-dorsal spine, at same height as posterio-dorsal spine, in lateral view. Ventro-lateral spine anterior to lateral spine (Fig. 22A), almost on ventral side of exuviae (Fig. 21B). Ventral spine very small, indistinct (Fig. 21C).

**Female pupa**. Acute dorsal spines of last abdominal segment similar to those of male (Fig. 21D, E and Fig. 22B). Cercus sheath elongated, longer than posterio-dorsal spine and hypogynial valva sheath; curved ventrally, anal spine small, not prominent (Fig. 21D–F). Hypogynial valva sheath acute triangle, shorter than posterio-dorsal spine (Fig. 21E and Fig. 22B). Ventro-lateral spine situated slightly more dorsal than that of male (Fig. 21E and Fig. 22B).

#### Distribution

Japan: Honshu, Kyushu and Shikoku Islands.

#### Biology

**Larval habitat and biology**

*Holorusia mikado* larvae were collected from aquatic and semi-aquatic habitats. The larvae were found along the banks of mountain streams, waterfalls and in drainage ditches at the side of the road, where decaying litter accumulated and the water flow was relatively slow (Fig. 1). Larvae of Pedicia (Pedicia) spp. and Tipula (Platytipula) sp. were collected together with *H. mikado* in the same microhabitat (Oguni, Honshu Is.). The larvae of *Holorusia* are detritivores, feed on wet, decomposing leaves. They prefer thinner, softer leaves like maples (*Acer* spp.) over harder leaves. The habitat niche of the larvae is very similar to those of the Tipula (Acutipula) maxima Poda, 1761 species-group in the Western Palearctic Region. Both groups prefer banks of smaller streams where litter accumulates. The general appearance of larvae of the these two groups are also similar, as in the elongated anal papillae and the general shape of the spiracular field, with relatively long setae on the margin of the spiracular lobes. Both features are characteristics of semi-, to almost freely-aquatic tipulid larvae (Brindle 1957, Keresztes et al. 2018).

Larvae of aquatic Tipula (Nippotipula) are also known to occur and feed on decaying leaves (Gelhaus 1986). Jo (2017) described the immature stages of Tipula (Nippotipula) sinica Alexander, 1935 from South Korea, collected from different microhabitats in small streams. Corrigendum: The species is misidentified in Jo (2017) and the description of larvae and pupae refer to Tipula (Nippotipula) coquilletti Enderlein, 1912 and not to T. (N.) sinica (JaeIck Jo pers. comm.).

**Life cycle and activity**

**Figure 23.**
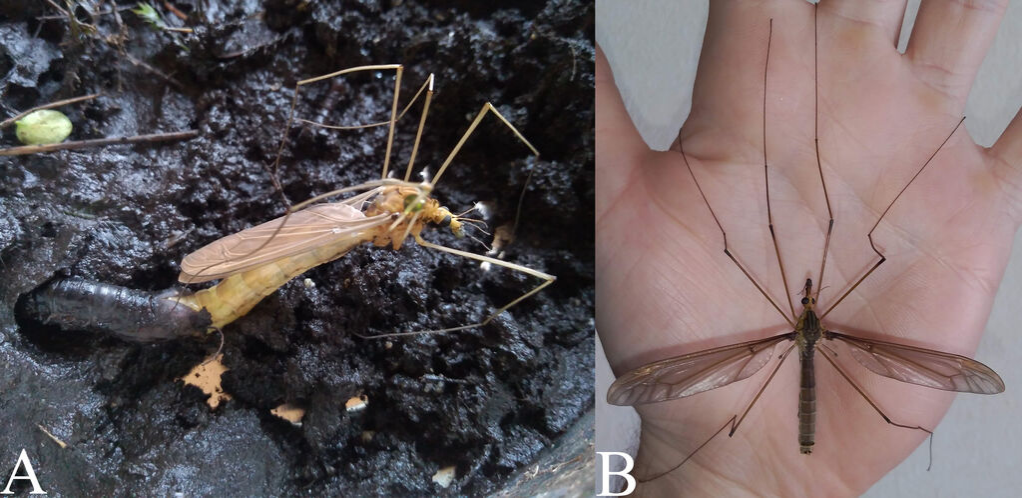
*Holorusia mikado* (Westwood, 1876). **A.** Female emerging from pupa; **B.** Freshly emerged male.

Four larvae of *Holorusia mikado* pupated almost in the same period, with about two and a half days between the earliest and latest pupations. Two males and two females emerged 7–8 days after the pupations, in the morning between 5 and 9 am. (Fig. 23A). The imagos were resting on vertical surfaces during the day and outspread their wings horizontally (Fig. 23B). They were active (flew around) in the insect cage (BugDorm) from the afternoon to midnight. Copulation was not observed. One female was kept separately in a different cage, it laid 70–90 unfertilised eggs to the sieved wall of the cage within three hours. The eggs were deformed and did not stick to any surfaces (Fig. 6E). The female died soon after laying the eggs.

**Flying period of imagos**: Early May to late August.

#### Taxon discussion

**Larva**

The last instar larvae of *H. mikado* and *H. hespera* differ, particularly in the number of anal papillae and the pattern of the spiracular field. *H. hespera* has three-three lateral anal papillae, while *H. mikado* has an additional unpaired lobe between the lateral papillae, which is directed anteriorly (Fig. 18B and C). The odd number of anal papillae of *H. mikado* is quite a unique character within Tipuloidea as crane flies larvae usually have an even number of anal papillae. This is the first known Tipulidae species having odd-numbered anal papillae. The delicate black line on the inner surface of the dorsal lobes are barely indicated in *H. hespera*, while it is dark and clearly noticeable in *H. mikado*. Furthermore, the median black line on the ventral lobe is short in *H. mikado* and does not reach the base of the lobe (Fig. 17), while it is long in *H. hespera* and almost reaches the base of the lobe (see Alexander 1920 fig. 496 and Young 2004 fig. 2B). The hypopharynx is six-toothed in *H. hespera* (see Alexander 1920 fig 494) and five-toothed in *H. mikado* (Fig. 12D and E).

**Pupa**

The pupae of *H. hespera* and *H. mikado* differ in the length of their respiratory horns, which are 1.75 times longer than the width of the thorax in *H. mikado* and about 1.25 times longer in *H. hespera*. The mid-leg sheath is slightly longer than the hind-leg sheath in *H. hespera* and clearly shorter than the hind-leg sheath in *H. mikado* (Fig. 19E). Pleurites have one basal and three posterior spines in the case of *H. mikado*, but *H. hespera* has one basal and two posterior spines. The anal spine of the male genital sheath is slender in *H. hespera* (see Alexander 1920 fig. 497), but short and stout in *H. mikado* (*Fig. 21*B and Fig. 22A).

#### Notes

The identification of “the largest specimen of crane-fly” as “*Holorusia mikado*”, registered by the Guinness World Records from China (Sichuan) is very questionable, based on the photos that are published on the internet. The specimen has a whitish scutellum and mediotergite, which is greyish-brown on *H. mikado* specimens. The Chinese specimen obviously belongs to another *Holorusia* species; therefore, we removed this species from the Chinese checklist. Although the Catalogue of the Craneflies of the World also lists *H. mikado* from the Island of Taiwan (Oosterbroek 2020), we have not found any evidence that this species was reported from the Island. The species is not listed in the Catalogue of the Diptera of the Oriental Region (Alexander and Alexander 1973) and most probably, the record from Taiwan was accidentally listed in the Catalogue of Palaearctic Diptera (Oosterbroek and Theowald 1992) from Taiwan (Pjotr Oosterbroek, pers. comm.). The only publication that mentions the species and related to Taiwan (as Formosa) is Edwards (1921). The title of this publication was “New and little-known Tipulidae, chiefly from Formosa”; however, the localities of *H. mikado*, listed in the article, are all from the territory of present-day Japan. Despite long-term studies (second and third authors), we have never found *H. mikado* on the Ryukyu Islands, the Oriental Island chain lying between Kyushu and Taiwan. For these reasons, we also remove the record of *H. mikado* from Taiwan in this manuscript and consider that *H. mikado* occurs only on the Palaearctic Japanese Islands of Honshu, Shikoku and Kyushu, but not on Hokkaido.

## Supplementary Material

XML Treatment for Holorusia mikado
